# Olfactory Impairment Is Correlated with Confabulation in Alcoholism: Towards a Multimodal Testing of Orbitofrontal Cortex

**DOI:** 10.1371/journal.pone.0023190

**Published:** 2011-08-12

**Authors:** Pierre Maurage, Christophe Callot, Betty Chang, Pierre Philippot, Philippe Rombaux, Philippe de Timary

**Affiliations:** 1 Neuroscience, Systems and Cognition (NEUROCS) and Health and Psychological Development (CSDP) Research Units, Institute of Psychology, Catholic University of Louvain, Louvain-la-Neuve, Belgium; 2 Health and Psychological Development (CSDP) Research Unit, Institute of Psychology, Catholic University of Louvain, Louvain-la-Neuve, Belgium; 3 Department of Otorhinolaryngology, St Luc Hospital and Institute of Neuroscience, Catholic University of Louvain, Brussels, Belgium; 4 Department of Psychiatry, St Luc Hospital and Institute of Neuroscience, Catholic University of Louvain, Brussels, Belgium; University of Granada, Spain

## Abstract

**Background:**

Olfactory abilities are now a flourishing field in psychiatry research. As the orbitofrontal cortex appears to be simultaneously implicated in odour processing and executive impairments, it has been proposed that olfaction could constitute a cognitive marker of psychiatric states. While this assumption appears promising, very few studies have been conducted on this topic among psychopathological populations. The present study thus aimed at exploring the links between olfaction and executive functions. These links were evaluated using two tasks of comparable difficulty, one known to rely on orbitofrontal cortex processing (i.e., a confabulation task), and one not associated with this area (i.e., Stop-Signal task).

**Methodology/Principal Findings:**

Twenty recently detoxified alcoholic individuals and twenty paired controls took part in an experiment evaluating olfactory abilities and executive functioning (i.e., Stop-Signal task and confabulation task). Comorbidities and potential biasing variables were also controlled for. Alcoholic individuals exhibited impaired performance for high-level olfactory processing and significant confabulation problems as compared to controls (but no deficit in Stop-Signal task), even when the influence of comorbidities was taken into account. Most importantly, olfactory abilities and confabulation rates were significantly correlated in both groups.

**Conclusions/Significance:**

Alcoholism jointly leads to olfactory and memory source impairments, and these two categories of deficits are associated. These results strongly support the proposition that olfactory and confabulation measures both index orbitofrontal functioning, and suggest that olfaction could become a reliable cognitive marker in psychiatric disorders. Moreover, it underlines the need to take into account these olfactory and source memory impairments in a clinical context.

## Introduction

The importance of sensory and emotional processes in the development and maintenance of psychiatric disorders is now widely recognised. While most studies classically used visual and auditory stimulations, olfaction has recently received more attention, and several studies have described olfactory alterations in various psychiatric states such as autism [1], depression [2]-[4], dementia [5], anorexia nervosa [6], alexithymia [7] and schizophrenia [8], [9]. These observations have undeniably enriched the theoretical and clinical understanding of these conditions, notably by proposing that each disorder could be characterized by a specific impairment pattern of olfactory abilities [see 10 for a review]. Odour processing thus constitutes a topic of rising importance in psychiatry, first in view of the olfactory impairments' harmful consequences in everyday life [11], but also because the olfactory system is closely connected with brain areas responsible for the most crucial and explored alterations among psychiatric populations, namely executive and emotional impairments [12].

Indeed, olfaction is the only sensorial modality to possess direct connections with the limbic and fronto-temporal areas implicated in executive processing [13], [14]. More specifically, the orbitofrontal cortex constitutes a critical structure concerning this pathway as it is simultaneously implicated in the processing of olfactory and emotional/executive stimulation [15], [16]. It has been recently proposed that olfactory abilities could represent a cognitive marker of psychiatric states [17], [18], and that olfactory stimulation could constitute a new tool to explore the structural and anatomical dysfunctions of the brain regions (particularly the orbitofrontal cortex) associated with high-level cognitive functions. Nevertheless, that assumption has up to now only been explored in schizophrenia [19]-[22].

This lack of data concerning olfaction and its connections with executive functions in the orbitofrontal cortex is particularly apparent in alcoholism. Indeed, although alcohol dependence is the most widespread psychiatric disorder [23], very few studies have explored olfactory deficits among alcoholic inpatients, and they reported contradictory results [24]-[32]. Moreover, only one study [26] explored the potential links between olfaction and executive functions in alcoholism, leading to promising preliminary observations by showing that executive functions seem to be correlated with odour discrimination, but not with odour identification.

There is thus a discrepancy between the theoretical assumption of an olfactory-executive connection via the orbitofrontal cortex and the divergent experimental results obtained among psychiatric populations. This inconsistency could be partially explained by limitations of earlier studies. First, several studies did not control for interfering variables like comorbidities and medication, which can influence the results [33]. Secondly and most importantly, previous research used questionable executive measures as indexes of orbitofrontal functioning. Studies conducted in schizophrenia and alcoholism were based on paradigms (i.e., Wisconsin Card Sorting Test, Stroop test, Stop-Signal task) that are not specifically designed to explore orbitofrontal functioning, but that are rather known to involve a more diffuse frontal network including dorsolateral or mediofrontal cortices, which are not centrally involved in olfaction. Thus, the contradictory results of previous research could be due to the use of paradigms that are not well-targeted to explore orbitofrontal cortex.

Considering the limitations of past studies, the aim of the present study is to clarify the links between olfaction and executive functions among alcoholic and control individuals, by means of a paradigm specifically associated with orbitofrontal cortex functioning and with a strict control of potentially biasing variables. The confabulation task [34], specifically designed to test source memory impairment among confabulators, has been repeatedly shown to be associated with orbitofrontal cortex activation [35], [36]. Moreover, a frequent complication of alcohol dependence is the Korsakoff syndrome, which has been repeatedly shown to induce confabulations (i.e., the tendency to replace a gap in one's memory by a falsification that one believes to be true). It can thus be suggested that confabulation task could be impaired among alcohol dependent individuals, and we chose this specific task to test our main hypothesis in the following manner: If olfactory impairments are related to orbitofrontal dysfunction in psychiatry, they should be strongly correlated with deficits in the confabulation task among alcoholic participants. Moreover, in order to confirm that the potential correlation between confabulation and olfaction is indeed a specific index of orbitofrontal impairment and is not due to a more general cognitive disturbance, a classical frontal test (i.e., Stop-Signal task, [37], [38]) will be used as a control cognitive task. Indeed, this Stop-Signal task is usually not associated with significant orbitofrontal activations, but rather with dorsolateral prefrontal cortex and middle frontal gyrus activations [39]–[41].

## Methods

### Ethics Statement

Participants were provided with full details regarding the aims of the study and the procedure to be followed. After receiving this information, all participants gave their informed written consent. The study was approved by the Ethics Committee of the Medical School (Catholic University of Louvain) and was conducted according to the principles described in the Declaration of Helsinki.

### Participants

Twenty inpatients (nine women), diagnosed with alcohol dependence according to DSM-IV criteria, were recruited during the third week of their treatment in a detoxification center (St. Luc Academic Hospital, Catholic University of Louvain, Brussels, Belgium). They had all abstained from alcohol for at least two weeks (*M* = 15.23 days; *SD* = 2.94), were free of any other psychiatric or Korsakoff syndrome diagnosis as assessed by an exhaustive psychiatric and neurological examination (exclusion criterion was comorbidity with any other psychiatric or neurological disease, including head trauma and epilepsy) and were all right-handed. The mean alcohol consumption among alcoholic participants just before detoxification was 13.8 alcohol units (an alcohol unit corresponds here to 10 grams of pure ethanol) per day (*SD* = 10.18), the mean number of previous detoxification treatments was 2.15 (*SD* = 3.13), and the mean duration of alcohol dependence was 15.9 years (*SD* = 12.99). Alcoholic individuals were matched for age, gender and education level with a control group composed of 20 volunteers who were free of any history of psychiatric disorder or drug/substance abuse. The mean alcohol consumption among controls was 4.2 units per week (*SD* = 1.9), and they abstained from any alcohol consumption for at least three days before testing. Exclusion criteria for both groups included major medical problems, neurological impairment, positive history of olfactory loss or olfactory disorder, and polysubstance abuse. Education level was assessed according to the number of years of education completed since starting primary school. Nine alcoholic participants and eight controls presented nicotine dependence: The mean number of cigarettes per week was 128.7 among controls (*SD* = 78.9) and 94.3 among alcoholics (*SD* = 68.9), and the mean duration of smoking habits was 10.98 years among controls (*SD* = 12.82) and 15.44 years among alcoholics (*SD* = 12.46). Although all controls were free of any medication, eleven alcoholic individuals still received low doses of diazepam (mean among medicated inpatients: 25.5 mg/day; *SD* = 28.83).

### Task and procedure

#### A. Control measures

Every participant was assessed using validated self-completion questionnaires to evaluate the presence of sub-clinical psychopathologies: anxiety (State and Trait Anxiety Inventory, form A and B [42]), depression (Beck Depression Inventory [43]), alexithymia (20-item Toronto Alexithymia Scale [44]) and empathy (Empathy Quotient [45]).

#### B. Psychophysical testing of olfactory function

Two psychophysiological testings were proposed for olfactory function. Firstly, an *orthonasal testing*, in which orthonasal olfactory function was assessed by means of the standardized “Sniffin' Sticks” test [46]. In this evaluation, odours are presented using felt-tip pens containing a tampon filled with four millilitres of liquid odorants. During odour presentation, the experimenter removes the pen's cap and places the pen approximately two centimetres in front of both nostrils for three seconds. This test evaluates olfactory acuity on the basis of three subtests. The first subtest examined odour threshold, which was assessed with N-butanol using stepwise dilutions in a row of 16 felt-tip pens. The task was a triple-forced choice: Three pens were presented in a randomized order (two containing the solvent and the third the odorant at a certain dilution), and the participant had to identify the odour-containing pen. The odour threshold score ranged from 0 to 16. The second subtest examined odour discrimination, using 16 triplets of pens (two containing the same odorant and the third the target odorant) presented in a randomized order. Subjects had to identify which odour-containing pen smelled different from the two others. The odour discrimination score ranged from 0 to 16. The third subtest examined odour identification, which was evaluated using 16 common odours. Participants were asked to identify each odour using multiple-choice lists of four items. The odour identification score ranged from 0 to 16. Finally, results for odour threshold (T), odour discrimination (D), and odour identification (I) were summarized in a composite threshold-discrimination-identification (TDI) score, ranging from 0 to 48.

Secondly, a retronasal testing in which retronasal olfactory function was evaluated using a standardized and validated test [47] based on the identification of 20 odorized powders or granules presented in the oral cavity. Stimulants were applied to the midline of the tongue. For each item, participants were asked to perform a forced choice from a list of four items. Participants rinsed with water after each powder was administered. The percentage of correct response was calculated based the 20 items.

#### C. Executive function measures

Two experimental tasks were conducted to evaluate executive functions. Firstly, a *Stop-Signal task*, which was a computer adaptation of the classical Stop-Signal task [37], [38], which involves the inhibition of a previously learnt and prepotent categorization response. In a first block (48 trials), participants were asked to decide whether the word presented on the computer screen was an animal name or an object name, which they did by pressing a corresponding button. Twelve animal names and 12 objects names, controlled for length and frequency, were randomly and successively presented (two times each) on the computer screen for 1500ms. In a second block (192 trials), participants had to perform the same task with the same words, except when a sound (i.e., a beep lasting for 400 ms) was emitted by the computer just after the appearance of the word. Thus, for these “stop” trials, participants had to inhibit the prepotent categorization response. Thirty-four practice trials were presented before the first block. Participants were told that they had to respond as quickly as possible without waiting for the possible appearance of the beep. If a slowing down was detected, the experimenter reminded the participant to respect this instruction. The time interval between stimulus and beep onset in stop trials was individually calculated and corresponded to the mean reaction time in the first block for the participant minus 300 ms. Each trial comprised a 500ms fixation point (white cross in the centre of a black screen) followed by the word for 1500 ms. Participants had thus 1500 ms to answer after stimulus onset. Reaction times and categorization error percentages were recorded for both blocks, but the critical inhibition measure was the percentage of categorization responses for the stop trials. Secondly, a *Confabulation task*, adapted from the confabulation paradigm [34]. The confabulation task was based on a continuous recognition paradigm and divided into two blocks. The first block was a simple item storage and recognition task. It comprised six trials, each presenting a sequence of 20 black-and-white drawings of real objects or animals. Each sequence contained eight target drawings and 12 distracter drawings presented one by one. The same eight targets appeared in each sequence, but the distracters were never repeated. There were 120 pictures in total. Upon seeing each picture, participants had to decide whether it was previously presented in the current block, by pressing the corresponding button. Each trial comprised a 700 ms fixation cross followed by a drawing which stayed on the computer screen until the participant responded. Twenty practice trails (3 targets) were presented before the first block, using pictures different from those used in the main task. The second block, presented one hour after the first one, used exactly the same procedure and stimuli. But in this block target items were replaced so that eight distracters from the first block were now the target items, while the target items from the first block were now distracters. Participants still had to decide whether each picture had already been presented in the current block. Importantly, they were asked to forget the items presented in the first block, so that items presented for the first time in the second block (but which were already presented in the first block) could erroneously be considered as targets by the participants. False positive answers in the second block (i.e., considering a new item as one already seen) could thus be based on an inability to distinguish between the item's previous occurrence in the first rather than the second block. Reaction times, percentage of correct hits and false alarms were recorded within each block. The main confabulation measure was the temporal context confusion (TCC) index, defined as the relative increase of false positives in the second block as compared to the first (see [34] for details). This TCC index was computed using the following formula: TCC  =  (FP2/Hits2) - (FP1/Hits1), where FP1 and 2 represent the number of false positive in blocks 1 and 2 respectively, and Hits1 and 2 represent the number of correct hits in blocks 1 and 2 respectively.

#### D. Statistical analyses

To compare performance in measures of olfactory and executive (Stop-Signal and confabulation tasks) functions between groups, standard z-scores were first computed for each measure in each task (namely threshold, discrimination, identification, global orthonasal and retronasal scores for olfaction/reaction times, performance and Stop/TCC index for executive tasks). Then, omnibus repeated-measures MANOVA were computed separately for olfaction, Stop-Signal and confabulation tasks with measures as within-subjects factors and group as between-subjects factor. Significant multivariate and interaction effects were followed by univariate contrasts (post-hoc independent samples t-tests). Independent samples t-tests were also computed to explore group differences on control measures. Analyses of covariance (ANCOVA) were performed to test the influence of potential biasing variables. Finally, two-tailed Pearson correlations were used to explore the links between the different experimental data.

## Results

### Control measures

As illustrated in Table 1, groups did not significantly differ in age (*t*_38_ = 0.73, ns), education level (*t*_38_ = 0.24, ns), number of cigarettes smoked (*t*_15_ = 1.52, ns), duration of nicotine dependence (*t*_15_ = 1.93, ns), state anxiety (*t*_38_ = 0.88, ns), alexithymia (*t*_38_ = 1.37, ns) and empathy (*t*_38_ = 0.97, ns). Nevertheless, alcoholism was associated with significantly higher scores for depression (*t*_38_ = 2.8, *P*<0.01) and trait anxiety (*t*_38_ = 3.01, *P*<0.01).

**Table 1:** Alcoholic and control individuals' characteristics: mean (*S.D.*).

	Controls (N = 20)	Alcoholics (N = 20)
**Age** ^ NS^	47.75 (*9.73*)	50.25 (*11.79*)
**EL** 1, NS	15.4 (*3.18*)	15.15 (*3.36*)
**BDI** 2, *	3.7 (*4.38*)	9.28 (*7.63*)
**Stai A** 3, NS	42.6 (*11.46*)	46.11 (*13.38*)
**Stai B** 3, *	43.6 (*9.21*)	53.58 (*11.46*)
**TAS-20** 4, NS	44.55 (*10.84*)	49.11 (*9.46*)
**EQ** 5, NS	38.6 (*6.24*)	40.3 (*14.32*)

NS  =  non-significant; *p<0.01.

1 EL  =  Education Level (in years).

2 BDI  =  Beck Depression Inventory (Beck & Steer, 1987).

3 STAI  =  State and Trait Anxiety Inventory (Spielberger et al., 1983).

4 TAS-20  =  Twenty-item Toronto Alexithymia Scale – II (Bagby et al., 1994).

5 EQ  =  Empathy Quotient (Baron-Cohen & Wheelwright, 2004).

### Psychophysical olfactory measures

The 5X2 MANOVA on olfaction performance [with measures as within-subjects factors (i.e., threshold, discrimination; identification, global TDI and retronasal scores) and group as between-subjects factor (i.e., control and alcohol-dependent participants)], showed a significant main effect of group (*F*_1,38_ = 24.23, *P*<0.001) and a group-by-measure interaction (*F*_4,152_ = 3.41, *P*<0.05). Post-hoc t-tests showed no significant group differences concerning odour detection threshold (*t*_38_ = 0.66, ns), but alcoholic participants obtained significantly lower scores than controls for odour discrimination (*t*_38_ = 2.08, *P*<0.05), odour identification (*t*_38_ = 4.03, *P*<0.001), TDI global orthonasal score (*t*_38_ = 3.71, *P*<0.001) and retronasal score (*t*_38_ = 4.57, *P*<0.001).

### Executive functions measures

Concerning the Stop-Signal task, the 5X2 MANOVA [with measures as within-subjects factors (i.e., reaction times and performance for blocks 1 and 2, and Stop-Signal index) and group as between-subjects factor (i.e., control and alcohol-dependent participants)], showed a significant group-by-measure interaction (F_4,152_ = 4.12, P<0.05) but no main effect of group (F_1,38_ = 1.52, ns). Post-hoc t-tests showed that groups did not significantly differ in their performance for the first (t_38_ = 1.37, ns) and second (t_38_ = 1.94, ns) blocks, nor for the Stop-Signal Index (i.e., percentage of categorization responses for stop signals, t_38_ = 0.74, ns), but alcohol-dependent participants obtained longer reaction times than controls for blocks 1 (t_38_ = 2.76, P<0.01) and 2 (t_38_ = 2.36, P<0.05).

Concerning the confabulation task, the 7X2 MANOVA [with measures as within-subjects factors (i.e., reaction times, hits and false positive for blocks 1 and 2, and TCC index) and group as between-subjects factor (i.e., control and alcohol-dependent participants)], showed a significant main effect of group (F_1,38_ = 8.28, P<0.01) and a group-by-measure interaction (F_6,218_ = 2.63, P<0.05). Post-hoc t-tests showed no group differences for the number of hits at block 1 (t_38_ = 0.99, ns) and 2 (t_38_ = 0.83, ns), nor for the percentage of false positives in block 1 (t_38_ = 0.76, ns). Nevertheless, alcoholics presented significantly delayed reaction times for block 1 (t_38_ = 3.13, P<0.01) and 2 (t_38_ =  2.27, P<0.05), as well as a higher number of false positives for block 2 (t_38_ =  2.26, P<0.05) and a higher TCC index (t_38_ =  2.71, P = 0.01) as compared to controls. Experimental results are illustrated in Table 2.

**Table 2:** Alcoholic and controls individuals' results for experimental measures: mean (S.D.).

	Controls (N = 20)	Alcoholics (N = 20)
**Olfaction**		
Odor Threshold score (0-16) ^NS^	5.82 (0.67)	5.65 (0.97)
Odor Discrimination score (0-16) *	13 (1.68)	11.8 (1.96)
Odor Identification score (0-16) ***	12.75 (0.91)	10.85 (1.89)
TDI Global score (0-48) ***	31.5 (2.05)	28.25 (3.46)
Retronasal Testing (% correct) ***	72.1 (9.77)	56.1 (12.18)
**Executive functions**		
* Stop Signal Task*		
Performance Block 1 (% correct) ^NS^	95.89 (3.39)	93.78 (5.97)
Reaction Times Block 1 (ms) **	606 (71.8)	717 (164.4)
Performance Block 2 (% correct) ^NS^	91.41 (6.97)	83.65 (16.21)
Reaction Times Block 2 (ms) *	705 (89.12)	798 (150.7)
Stop Signal Index ^NS^ (% resp. to stop trials)	27.9 (18.62)	32.7 (22.17)
*Confabulation Task*		
Number of Hits Block 1^ NS^	39 (0.85)	38.55 (1.84)
Number of False Positive Block 1^ NS^	3.15 (2.74)	2.55 (2.26)
Reaction Times Block 1 (ms) **	786 (122.8)	1008 (285.3)
Number of Hits Block 2^ NS^	36.37 (2.49)	37.05 (2.61)
Number of False Positive Block 2 *	3.89 (2.64)	7.85 (7.17)
Reaction Times Block 2 (ms) *	774 (118.9)	932 (282.1)
Temporal Context Confusion Index **	0.032 (0.071)	0.145 (0.167)

NS  =  non-significant;

*p<0.05 ;

**p<0.01 ;

***p<0.001

### Correlations between olfactory and executive function measures

In order to test the hypothesis that olfaction and executive functions could be associated, and that both index orbitofrontal cortex functioning, Pearson's correlations (calculated across groups and within each group) were computed between olfactory and executive function measures. The correlations between olfactory performance and Stop-Signal task results did not reach significance, but a consistent pattern of significant negative correlations was found between the confabulation task and olfactory performance. Indeed, TCC index score was significantly correlated in both groups with high-level olfactory tasks, namely odour identification, TDI global score and retronasal testing. These results are shown in Table 3.

**Table 3:** Pearson's correlations in both groups (N = 20) between olfactory results (horizontal) and executive functions data (vertical): ρ value (*P-value*). Significant results are indicated in bold text.

		Odor Threshold	Odor Discrimination	Odor Identification	TDI Global Score	Retronasal Testing
**SSI** 1	Controls	-0.35 *(N.S.)*	-0.02 *(N.S.)*	0.14 *(N.S.)*	0.04 *(N.S.)*	0.11 *(N.S.)*
	Alcoholics	-0.15 *(N.S.)*	-0.33 *(N.S.)*	-0.02 *(N.S.)*	-0.13 *(N.S.)*	-0.22 *(N.S.)*
**TCC** 2	Controls	0.13 *(N.S.)*	-0.27*(N.S.)*	**-0.57 ** ***(p<0.01)***	**-0.52 ** ***(p<0.05)***	**-0.35 ** ***(p<0.05)***
	Alcoholics	-0.12 *(N.S.)*	-0.19 *(N.S.)*	**-0.62 ** ***(p<0.001)***	**-0.48 ** ***(p<0.05)***	**-0.32 ** ***(p<0.05)***

1 SSI  =  Stop Signal Index (percentage of categorization response to stop trials).

2 TCC  =  Temporal Context Confusion Index (FP2/Hits2) - (FP1/Hits1).

### Complementary analyses

#### Complementary analyses were computed in order to

(1) Test for gender and age effects: these variables were included as covariates in our ANOVA statistical analyses. We did not observe any significant influence of gender or age on any experimental result (*F*_1,36_<0.62, *P*>0.43 for every test).

(2) Test the influence of psychopathological scores, nicotine dependence and medication on experimental results. Pearson's correlations (calculated within each group and across groups) were computed between questionnaires scores, smoking habits characteristics and medication on one hand, and experimental results (olfaction and executive functions) on the other hand. No significant correlations were found (ρ<0.28, *P*>0.08). Moreover, these variables were included as covariates in our ANOVA statistical analyses. We did not observe any significant influence of psychopathological scores (*F*_1,31_<1.71, *P*>0.2 for every test), smoking habits (*F*_1,13_<0.68, *P*>0.41 for every test) or medication (*F*_1,17_<0,25, *P*>0.62 for every test) on any experimental result.

## Discussion

The main objective of the present study was to evaluate olfactory and executive functions among alcoholic individuals and to explore their correlations, in order to reinforce the recent observation that these two abilities are an index of orbitofrontal functioning [48]-[56], and to explore the proposition, raised by recent studies [17], [18], that olfaction could constitute a cognitive marker of psychiatric states.

Concerning olfaction, data showed a specific deficit for high-level olfactory abilities in alcoholism. Olfactory processing can be separated into a primary “sensory” level, indexed by odour detection threshold, and a secondary “cognitive” level, indexed by odour discrimination and identification [57]. The preserved odour detection threshold observed here, combined with impaired odour discrimination, odour identification and TDI score suggests that alcoholism does not lead to a general olfactory deficit, but rather to a specific impairment for high-level olfactory processing. Nevertheless, as our olfactory results could be partly explained by a difficulty effect (detection threshold being a simpler task than discrimination and identification), future studies will have to confirm this proposition of a specific impairment for high-level olfaction. We also showed for the first time that alcoholism is associated with retronasal olfactory impairment, which sheds new light on olfaction deficits in alcoholism as orthonasal and retronasal abilities rely on largely separate processes [58].

Concerning executive functions, the delayed reaction times in alcoholics for both parts of the Stop-Signal and confabulation tasks are in line with earlier studies showing a global perceptual and motor slowing down in alcoholism [59]. More centrally, the present study is the first to specifically explore confabulations among non-demented alcoholics, and the observation that alcoholics present a significantly higher TCC index than paired controls constitutes the first description of a confabulation problem among non-demented alcoholics. That confabulation problem cannot be explained by a more general cognitive impairment, by a difficulty effect or by an inability to correctly perform the task among alcoholics. Firstly, the groups did not differ in their hit rate in both blocks of the confabulation task, nor in their false alarms rates in the first block: This suggests that the memory deficit in alcoholics is specifically due to an increase in false alarms rates in the second block of the experiment, which typically indexes source memory impairment [60]. Secondly and more centrally, the fact that there were no group differences in the performance for the control executive task (i.e., the Stop-Signal task) allowed us to exclude the possibility that worse performance from alcoholic participants on the confabulation task was due to a general impairment of cognitive abilities. Finally, complementary analyses suggested that the deficits in olfactory abilities and source memory appear specifically associated with excessive alcohol consumption and not with alternative variables.

Still, the main result of the present study is that high-level olfactory functions are positively correlated with executive functions that implicate the orbitofrontal cortex (as indexed by source memory deficits), among alcoholics as well as control participants. This result among control participants is consistent with previous studies [61]-[63] showing that olfactory and executive functions are also associated in normal brain functioning. Although there was no association between odour discrimination and source memory deficits in our study, strong and coherent negative correlations were found between high-level olfactory functions scores (mainly odour identification, but also TDI global score and retronasal testing) and TCC index, showing a robust association between high-level olfactory impairment and source memory dysfunction. These results are in line with previous ones [26] describing a positive correlation between olfactory and executive abilities in alcohol dependence, and extend them by (1) specifying which executive functions (i.e., memory source) are strongly associated with olfaction; (2) showing that they are also present for control participants. It should also be noted that no significant correlations were found between olfactory measures and the Stop-Signal task. This shows the confabulation-olfaction links are selective and do not reflect a general association between olfaction and cognitive tasks. Nevertheless, while several previous neuroimaging studies have shown that the orbitofrontal cortex is implied in olfactory [48], [50], [52], [56] and memory source [49], [51], [53]-[55] processing, thus reinforcing the proposition that our tasks specifically explored orbitofrontal functions, the present study was not based on neuroimaging data and future studies will thus have to confirm the present results by means of neuroimaging explorations.

These data support the proposition of the importance of the orbitofrontal deficits in the development and maintenance of psychiatric syndromes, and confirm that these deficits can be assessed by high-level olfactory and memory source measures [64]. This observation is congruent with earlier ones obtained among schizophrenic patients [19], [20]: Frontal cortex functioning can be jointly explored in psychiatry by means of olfactory and executive tasks.

Nevertheless, the present study has several limitations. First, as the alcohol dependent participants had only been abstinent for two weeks, it can not be totally excluded that residual acute effects of alcohol ingestion could have influenced the results. The effects observed here should thus be replicated among participants with longer abstinence duration. Second, only two cognitive tasks were used in this study (i.e. confabulation task to index orbitofrontal functioning, and Stop-signal task as a non-orbitofrontal control task). While the confabulation task is usually considered as indexing orbitofrontal functioning, several studies failed to show this association [65], [66]. Conversely, some studies showed marginal orbitofrontal activation during Stop-Signal task [67]. The tasks used here can thus not be considered as totally pure, and the present results should be confirmed and extended by using a broader range of cognitive tasks specifically associated with orbitofrontal cortex, e.g. object alternation task [68].

Despite these limits, the present results bare several implications. At the theoretical level, they suggest that olfaction might play a role in alcoholism, particularly as an index of executive impairment. The present data thus call for future studies to further explore this sensorial modality. They also emphasize the usefulness of a multimodal exploration of the impairments exhibited by psychiatric inpatients, by showing the complementary data provided by olfactory and source memory testing for the understanding of orbitofrontal functioning. At the clinical level, our results suggest that orbitofrontal impairments might have a deleterious effect on alcoholics' everyday functioning. On one hand, orbitofrontal impairments are implicated in olfactory dysfunction, which in turn plays a role in the nutritional problems and decline in quality of life observed in alcoholic individuals [69], [70]. On the other hand, orbitofrontal impairments underlie source memory deficits, which are known to compromise daily functioning [71], [72]. Source memory and olfactory deficits are currently ignored in clinical practice, and the present study underlines the importance of developing standard evaluation and specific therapeutic programs focusing on olfactory and source memory rehabilitation in alcoholism.
